# Standardizing the measurement and classification of quality of life using the Keratoconus End-Points Assessment Questionnaire (KEPAQ): the ABCDEF keratoconus classification

**DOI:** 10.1186/s40662-022-00288-0

**Published:** 2022-05-08

**Authors:** Kepa Balparda, Tatiana Herrera-Chalarca, Melissa Cano-Bustamante

**Affiliations:** 1 Department of Cornea and Refractive Surgery, Black Mammoth Surgical, Carrera 43 # 29 - 35, Office 712, Medellín, Colombia; 2 Department of Clinical Research, Black Mammoth Surgical, Medellín, Colombia; 3 School of Medicine, Universidad Pontificia Bolivariana, Medellín, Colombia

**Keywords:** Quality of life, Keratoconus, Vision

## Abstract

**Background:**

Measuring quality of life is of importance in keratoconus. So far, the Keratoconus End-Points Assessment Questionnaire (KEPAQ) is the only keratoconus-specific scale to measure emotional well-being along with functional compromise in this population. Nevertheless, there is still a lack of clarity and standardization as to how KEPAQ scores should be computed and reported. There are also no guidelines for interpretation of classification of quality of life when using this instrument. The purpose of this study is to provide a specific framework on how to grade and classify keratoconus by using the KEPAQ and propose an extension of current classification to encompass subjective compromise.

**Methods:**

A group of patients with a confirmed diagnosis of keratoconus underwent application of the KEPAQ. First, a Rasch modeling was performed to evaluate the psychometric characteristics of both sub-scales of the instrument. Then, a linear transformation was performed to turn data into a more relatable scale ranging from 0 to 100. Finally, by using Tukey’s Hinges, scores of the KEPAQ were divided in a 1-to-4 scale, allowing for an easy E&F classification system.

**Results:**

A total of 386 applications of the KEPAQ were included. Analysis provided evidence of the KEPAQ being unidimensional, well-fitted to the Rasch Model, and provided adequate interval-level scores. Linear transformation resulted in a user-friendly final score ranging from 0 to 100, where a higher score translates to having a better quality of life. Two methods of easily computing final score, one by hand and the other one by an Excel file, were constructed. An E&F 1-to-4 classification was proposed, which may work well with the current ABCD classification of keratoconus.

**Conclusions:**

The KEPAQ is a psychometrically robust scale, which confidently measures both emotional-related and functional-related quality of life in patients with keratoconus. It can be easily computed, and the results are interpretable and classified in a manner similar to that used in the ABCD keratoconus classification, by adding letters ‘E’ for emotional and ‘F’ for functional compromise.

## Background

Measuring quality of life in ophthalmology is of utmost importance for both the researchers and clinicians alike, as it provides important information that cannot be completely obtained by measuring other variables, such as visual acuity and corneal distortion [1]. Whenever available, the researchers and clinicians should use disease-specific scales, as these will report more accurately than general-purpose scales in the specific studied population. So far, only two keratoconus-specific scales have been developed and validated through Rasch modeling: the Keratoconus Outcomes Research Questionnaire (KORQ) developed by Khadka et al. [2] and the Keratoconus End-Points Assessment Questionnaire (KEPAQ) developed by our group [1].

Although we have demonstrated that the KEPAQ complies with the stringent expectations of the Rasch model [1], and correlates well with corneal distortion [3], there is still some uncertainty regarding the best way to calculate, report and interpret results from the scale, especially for non-Rasch experienced researchers. Prior published papers have suggested calculating KEPAQ score by a raw-score-to-Rasch-measure table [1], but it is clear that this is not optimal as it does not allow for calculation when there are missing items. Moreover, it does not consider the relative weights of different questions while calculating the final score, which is one of the main hallmarks of Rasch modeling [4]. Besides, there may be some room for implementing a quality-of-life classification based on the KEPAQ, which may allow the clinicians to obtain a more complete picture on the way keratoconus affects the patient’s daily life.

The following paper provides a standardization for the way KEPAQ results should be computed and reported. It uses a linear transformation to turn Rasch non-user friendly logit results into an easily relatable scale ranging from 0 to 100, with a lower score translating to a lower quality of life. It provides two different methods for computing scores by the non-Rasch expert clinician: a “by hand” computation by means of tables that account for the differential weights of items towards the final score and allows for computation even when missing data exists; the other is an Excel file that allows for convenient real-time calculation of the final score. Finally, the article suggests a way in which results from the KEPAQ may be interpreted based on Tukey’s Hinges, and how this classification could be implemented to improve current Belin ABCD classification for the disease [5].

## Methods

The retrospective nature of this study is categorized as “non-risk investigation” following current Colombian law. Signed informed consent was also deemed unnecessary by the Ethical Committee.

The tenets of the Helsinki Declaration were adhered to and the research protocol was evaluated and approved by the Research Ethical Committee at the Clínica de Oftalmología Sandiego (Medellín, Colombia) under reference number “Keratoconus-01”.

This retrospective analytical study using Rasch modeling sought to provide a normative database on the results of the KEPAQ in keratoconus patients to allow for a standardization in the reporting, measuring and classification of data obtained from such scale.

### Study population

The authors retrospectively reviewed the charts of all keratoconus patients under the clinical care of the first author (K. B.) and who had previously undergone application of the final version of the KEPAQ [1]. If the same patient had undergone application of the KEPAQ more than once, all applications were taken into account as far as more than 2 months had passed between such applications. In case two applications had been performed earlier than 2 months apart, only the first of them was taken into account. A total of 386 different applications of the scale were found and used for analysis. Only patients with an age equal or greater than 15 years old were included.

Keratoconus was diagnosed based on the requirement of the global consensus on keratoconus and ectatic diseases, inclined appearance of posterior corneal elevations, an abnormal distribution of corneal thickness, corneal thinning and an increase in corneal curvature. Tomographic diagnosis was aided by a pathological Belin/Ambrosio display along with the Pentacam tomographic indices.

### KEPAQ scale

The KEPAQ is a self-administered, keratoconus-specific scale, recently developed and validated by our group [1]. It consists of a total of 16 questions divided into two sub-scales that measure different constructs. The first part of the scale comprises seven questions and evaluates the emotional compromise of the patients secondary to the disease (KEPAQ-E, Table 1). The second sub-scale comprises nine questions revolving around the functional compromise secondary to ectasia (KEPAQ-F, Table 2). All questions are written in a clear and concise manner and these aim to determine how much the patient feels the disease handicaps them in several different situations. All questions use a Likert-Like response system with a corresponding scoring system as follows: “Not at all” = 3; “A little” = 2; “Quite a bit” = 1; “A lot” = 0. All patients are also given the choice to select “Not applicable” if they feel the question does not correlate with any situation in their daily lives. Early tables for converting the total sum score into a non-transformed Rasch-derived score were previously published by our group [1, 6], with a greater KEPAQ score meaning less disability by disease [3]. A complete description and explanation on the origin and philosophy behind the development of the KEPAQ is available here [1]. Our group has previously demonstrated the KEPAQ to comply adequately to the Rasch model [1], to be unidimensional and reliable [6] and to correlate well with the Belin ABCD classification [3].

**Table 1:** Emotional compromise sub-scale of the Keratoconus End-Points Assessment Questionnaire (KEPAQ-E)

Code	Question	Not at all	A little	Quite a bit	A lot	N/A
Q_E01	Do you feel your eye disease has affected your confidence to perform your daily tasks?	3	2	1	0	X
Q_E02	Do you feel your eye disease has affected your confidence to leave the house?	3	2	1	0	X
Q_E03	Do you feel your eye disease has affected your happiness in general?	3	2	1	0	X
Q_E04	Do you feel your eye disease has affected your confidence to go from one place to another?	3	2	1	0	X
Q_E05	Do you feel your eye disease has affected your self-esteem?	3	2	1	0	X
Q_E06	Do you feel your eye disease has affected your confidence about the future?	3	2	1	0	X
Q_E07	Do you feel your eye disease has caused you fear about the future?	3	2	1	0	X

**Table 2:** Functional compromise sub-scale of the Keratoconus End-Points Assessment Questionnaire (KEPAQ-F)

Code	Question	Not at all	A little	Quite a bit	A lot	N/A
Q_F01	Do you feel your eye disease has affected your ability to play sports?	3	2	1	0	X
Q_F02	Do you feel your eye disease has affected your ability to see objects near you?	3	2	1	0	X
Q_F03	Do you feel your eye disease has affected your ability to perform your daily tasks?	3	2	1	0	X
Q_F04	Do you feel your eye disease has affected your ability to go to the movies?	3	2	1	0	X
Q_F05	Do you feel your eye disease has affected your ability to do your job?	3	2	1	0	X
Q_F06	Do you feel your eye disease has affected your ability to watch television?	3	2	1	0	X
Q_F07	Do you feel your eye disease has affected your ability to use the computer?	3	2	1	0	X
Q_F08	Do you feel your eye disease has affected your ability to read books?	3	2	1	0	X
Q_F09	Do you feel your eye disease has affected your ability to see objects that are faraway?	3	2	1	0	X

### Rasch modeling

Data obtained was studied through a Rasch modeling to thoroughly evaluate the KEPAQ’s psychometric properties and compliance with the model. Rasch modeling is currently regarded as the gold standard for modern psychometric evaluation [7, 8] and it has been clearly demonstrated to be superior to prior methods, now called classical test theory (CCT) [4]. An in-depth discussion on the characteristics and benefits of Rasch modeling is clearly outside the scope of our paper, and the interested reader is directed to the excellent works by Boone et al. [4, 7].

Data was analyzed in the following manner. First, all data was introduced into an ad-hoc database created for the project in Numbers version 10.3.5 build 7029.5.5 (Apple Inc; Cupertino, CA, United States). Then, it was exported into a comma-separated values (.csv) file and imported into Winsteps version 4.7.1 (J. M. Linacre; Beaverton, OR, United States) where two control files were created, one for every sub-scale, and each control file was studied independently. First, item polarity through point-measure correlation was checked as a means of diagnosing potential mistakes in the database. Then, scale quality parameters were evaluated, including a person separation (which is expected to be over 2.0) [1] and person reliability (which is expected to be over 0.80 for good functioning scales) [9]. The floor-ceiling effect was calculated and was expected to be under 10% when summing up both extreme maximum and extreme minimum sum scores [9].

Next category function was evaluated to determine usage of the different categories and category ordering was determined by evaluating Andrich’s thresholds. A category probability plot was graphed trough IRT Illustrator version 2017.1 (Psychomeasurement Systems LLC; Charlottesville, VA, United States) to visually evaluate the behavior of the scales. In case any disordering of the categories was found, category collapsing was performed until adequate ordering was achieved [7]. Then, item calibration (measure) was obtained for all items as well as with their respective standard error. Item fitting to the Rasch model was measured by both the infit mean-square (MNSQ) and outfit MNSQ*.* Keeping up with Wright and Linacre’s recommendations [10], a fit value between 0.50 and 1.50 was considered of good clinical use, while values between 1.51 and 2.00 were considered poorly useful but non degrading of the score. Finally, fit levels over 2.01 were considered as potential degrading of the scale and carefully evaluated.

A principal component analysis (PCA) of standardized residual was performed following Boone’s recommendations [7], looking for a potential second dimension in the measurement. An eigenvalue for the first or second contrast of over 2.00 was considered evidence suggestive of a second or third dimension. If a second dimension was suggested, then the subsets of items representing the opposite poles of the factor were extracted and cross-plotted looking for significant perturbations on the measurements, as suggested by Linacre [11]. A critical decision was made regarding whether a potential secondary dimension was of concern.

A differential item functioning (DIF) was explored through the Mantel–Haenszel method for the following potential confounding variables: sex (male vs. female), keratoplasty (yes vs. no), corneal rings (yes vs. no) and corneal crosslinking (yes vs. no) [12]. For items with a significant DIF behavior, size effect with its 95% confidence interval was calculated.

Finally, a Wright map was graphed with jMetrik version 4.1.1 (Psychomeasurement Systems LLC; Charlottesville, VA, United States) for each of the evaluated sub-scales.

All analyses were performed on a MacBook Air computer running MacOS Catalina version 10.15.2 (Apple Inc; Cupertino, CA, United Stated). For running Winsteps (a Windows-native software), Windows 10 Home version 20H2 build 19,042.508 (Microsoft; Redmond, WA, United States) was emulated through a virtual machine created with Parallels Desktop 16 for Mac version 16.1.2 build 49,151 (Parallels Inc; Bellevue, WA, United States).

## Results

### Studied sample

A total of 386 patients were included. Mean age was 29.41 ± 9.98 years, and most patients (n = 234, 60.62%) were female. Upon evaluating their previous surgery history, 53 patients (13.73%) had had a keratoplasty in at least one of their eyes, while 189 patients (48.96%) had a history of corneal rings in at least one eye. Regarding history of corneal crosslinking and phakic intraocular lens implantation, the proportions were 33.94% (n = 131) and 4.92% (n = 19), respectively.

Mean maximum keratometry (Kmax) was 52.63 ± 7.18 D, while mean thinnest pachymetry was 474.58 ± 49.54 µm. Mean asphericity (Q) was − 0.57 ± 0.47.

### Emotional sub-scale (KEPAQ-E)

A total of 386 unique measurements on the KEPAQ-E were obtained, yielding adequate scale measures, with a person separation of 2.60 and a person reliability of 0.87. Active datapoints were 2687, accounting for 99.4% of potential responses. Only 0.6% of potential responses were answered as N/A. Only 22 patients obtained an extreme score, accounting for a floor and ceiling effect of 5.69%.

**Fig. 1:**
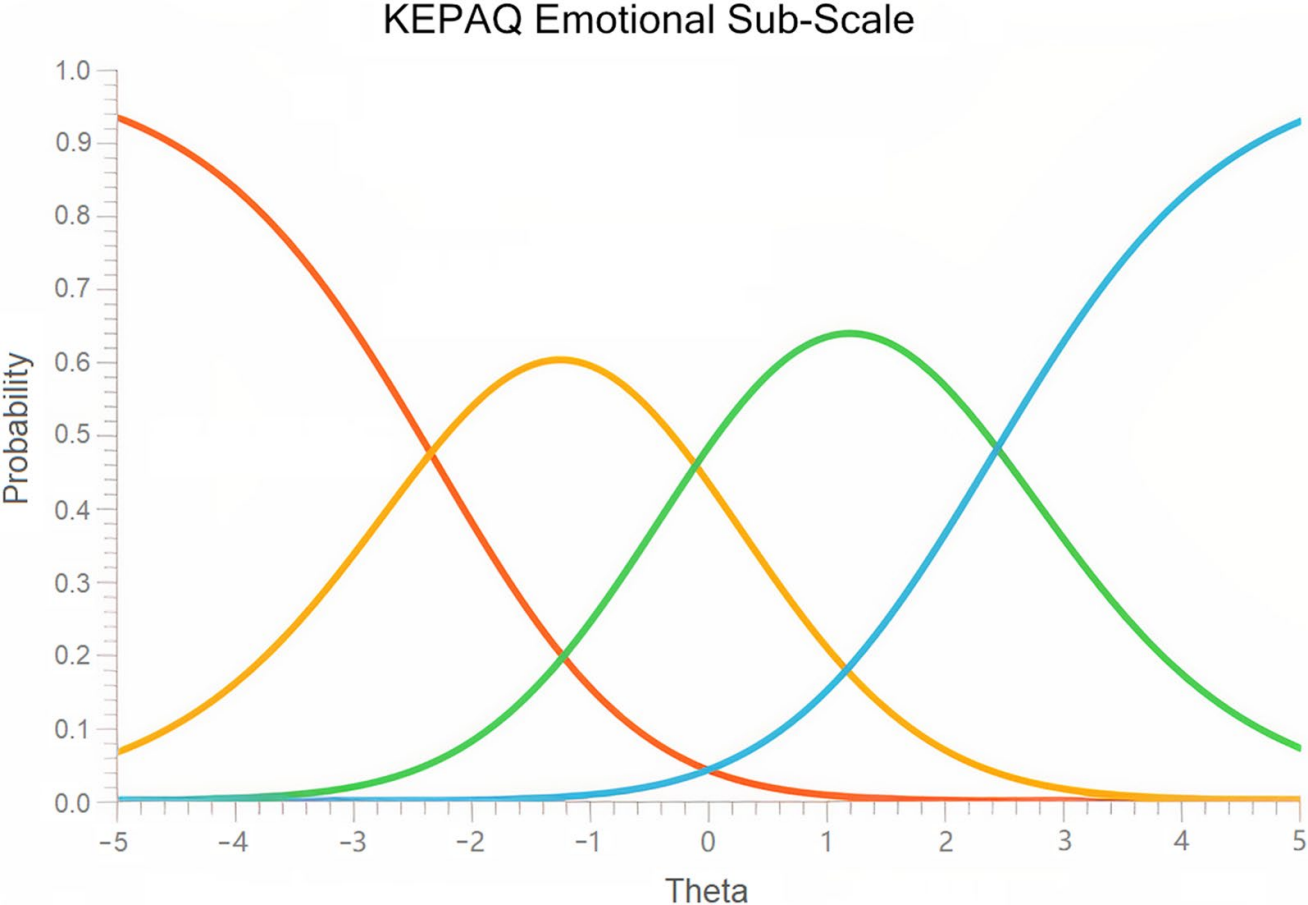
Category probability plot for the emotional compromise sub-scale of the Keratoconus End-Points Assessment Questionnaire (KEPAQ-E)

Item polarity ranged from 0.77 (Q_E07 fear about future) to 0.84 (Q_E04 place to another). Andrich’s threshold was shown to be well ordered (− 2.34; − 0.10; 2.45) and the category probability plot demonstrated well-functioning categories (Fig. 1). No category collapsing was deemed necessary. Item calibration ranged from − 1.14 logit (Q_E02 leave the house) to 1.73 logit (Q_E07 fear about future). No item was called to be either misfitting or overfitting (Table 3). Median person measure was 1.25 logit (interquartile range 3.65; skewness − 0.66; kurtosis 0.34; Kolmogorov-Smirnov *P* < 0.001).

**Table 3:** Item calibration and fitting for the emotional compromise sub-scale of the Keratoconus End-Points Assessment Questionnaire (KEPAQ-E)

Question	Measure	Infit MNSQ	Outfit MNSQ
Q_E01	− 0.17	0.97	0.92
Q_E02	− 1.14	1.03	0.78
Q_E03	− 0.53	1.12	1.11
Q_E04	− 0.78	1.00	0.83
Q_E05	− 0.73	1.08	1.06
Q_E06	1.63	0.92	0.99
Q_E07	1.73	1.05	1.10

PCA of the standardized residuals demonstrated that raw variance explained by measures was 66.9%. Eigenvalue for the first contrast was 2.57, explaining a variance of 12.2%. Two questions had a significant positive loading into a potential secondary dimension, Q_E06 confidence about future (loading 0.90) and Q_E07 fear about future (loading 0.88). A pilot separate analysis was performed by analyzing the KEPAQ-E but excluding Q_E06 and Q_E07, but the results obtained were not significantly different from what was obtained by analyzing all questions of the sub-scale together.

A DIF analysis was performed for sex (male vs. female), presence of keratoplasty (yes vs. no) and presence of corneal rings (yes vs. no), without finding any significant DIF in any item. DIF for presence or absence of corneal crosslinking, Q_E05 self-esteem, seemed to be slightly easier for the “presence of crosslinking” group (effect size 0.15; 95% confidence interval 0.02 to 0.28). All other items demonstrated a non-significant DIF behavior.

**Fig. 2:**
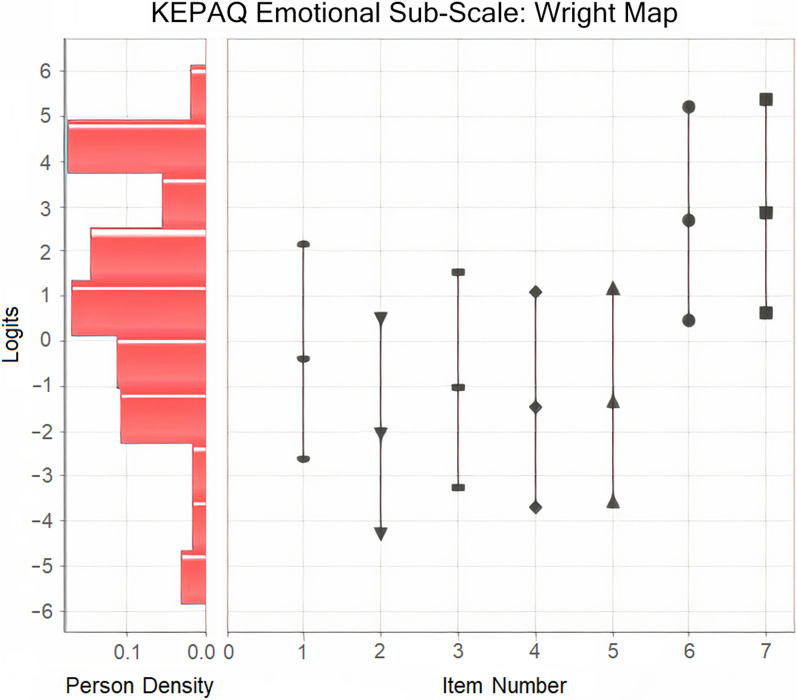
Wright map for the emotional compromise sub-scale of the Keratoconus End-Points Assessment Questionnaire (KEPAQ-E)

A Wright map was constructed for the emotional sub-scale (Fig. 2).

### Functional sub-scale (KEPAQ-F)

A total of 386 unique measurements on the KEPAQ-F were obtained, yielding adequate scale measures, with a person separation of 2.95 and a person reliability of 0.90. Active datapoints were 3441, accounting for 99.1% of potential responses. Only 0.9% of potential responses were answered as N/A. Only 36 patients obtained an extreme score, accounting for a floor and ceiling effect of 9.32%.

**Fig. 3:**
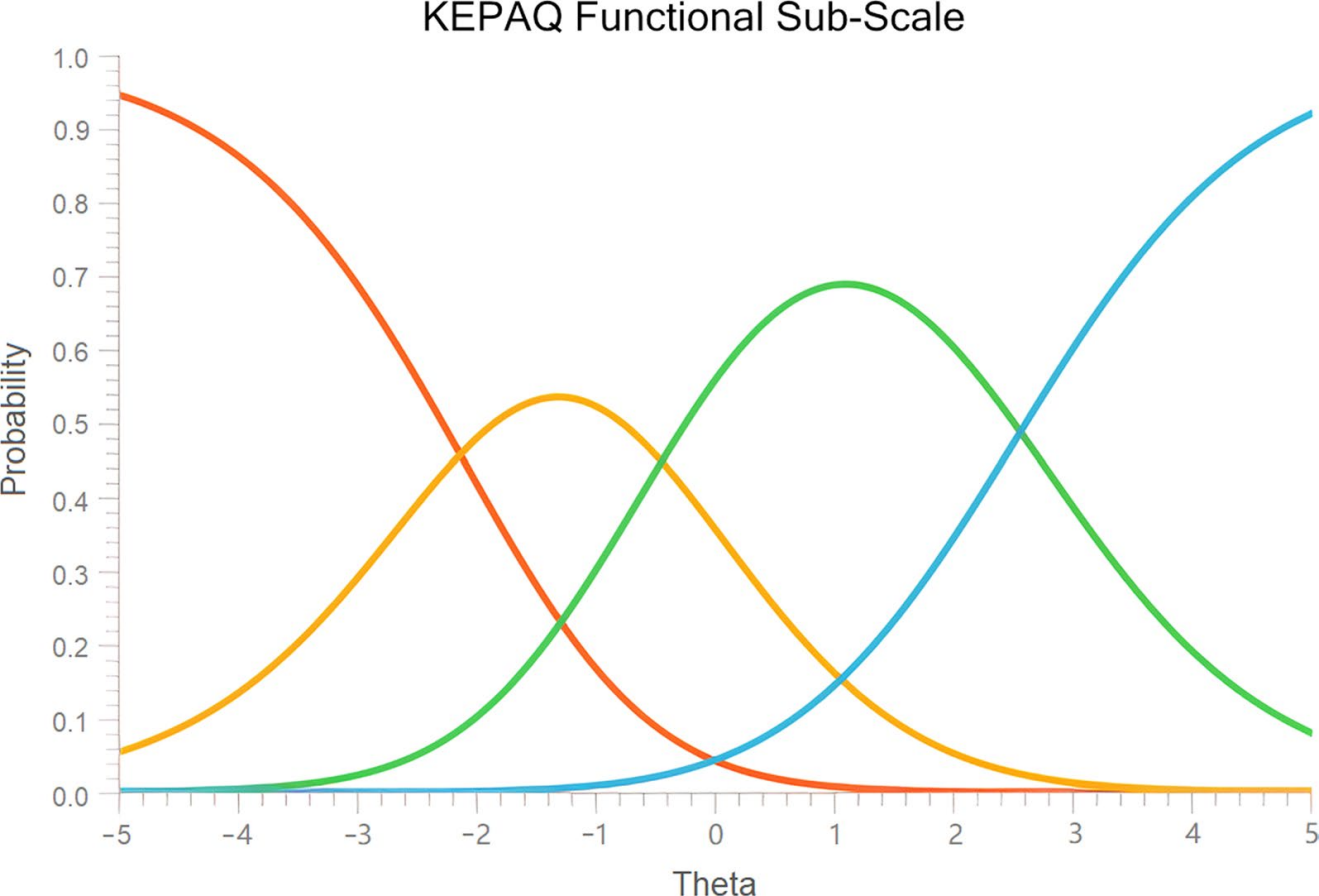
Category probability plot for the functional compromise sub-scale of the Keratoconus End-Points Assessment Questionnaire (KEPAQ-F)

Item polarity ranged from 0.69 (Q_F01 play sports) to 0.90 (Q_E04 watch television). Andrich’s threshold demonstrated to be well ordered (− 2.13; − 0.44; 2.57) and the category probability plot demonstrated well-functioning categories (Fig. 3). No category collapsing was deemed necessary. Item calibration ranged from − 1.03 logit (Q_F02 objects near) to 1.63 logit (Q_E09 objects faraway). Regarding fitting, Q_F01 play sports was found to have an infit MNSQ and outfit MNSQ over 2.0, therefore deemed to be somewhat misfitting and potentially degrading of the overall score. No other item was called to be either misfitting or overfitting (Table 4). A new analysis was performed for the KEPAQ-F excluding question Q_F01, but results were not different from questions that were already analyzed. Therefore, this question was not deemed to degrade measurement. Median person measure was 0.82 logit (interquartile range 3.88; skewness 0.09; kurtosis − 0.45; Kolmogorov-Smirnov *P* < 0.001).

**Table 4:** Item calibration and fitting for the functional compromise sub-scale of the Keratoconus End-Points Assessment Questionnaire (KEPAQ-F)

Question	Measure	Infit MNSQ	Outfit MNSQ
Q_F01	− 0.42	2.19	2.18
Q_F02	− 1.03	1.22	1.24
Q_F03	− 0.19	0.73	0.63
Q_F04	− 0.44	0.86	0.75
Q_F05	− 0.14	0.71	0.63
Q_F06	0.00	0.52	0.47
Q_F07	0.28	0.70	0.68
Q_F08	0.31	0.94	0.90
Q_F09	1.63	1.30	1.39

PCA of the standardized residuals demonstrated that raw variance explained by measures was 67.6%. Eigenvalue for the first contrast was 1.88, explaining a variance of 6.8%. Therefore, the scale was considered to be unidimensional.

A DIF analysis was performed for sex (male vs. female), presence of keratoplasty (yes vs. no) and presence of corneal rings (yes vs. no), without finding any significant DIF in any item. DIF for presence or absence of corneal crosslinking, Q_F07 use computer, seemed to be slightly easier for the “presence of crosslinking” group (effect size 0.18; 95% confidence interval 0.05 to 0.26). All other items demonstrated a non-significant DIF behavior.

**Fig. 4:**
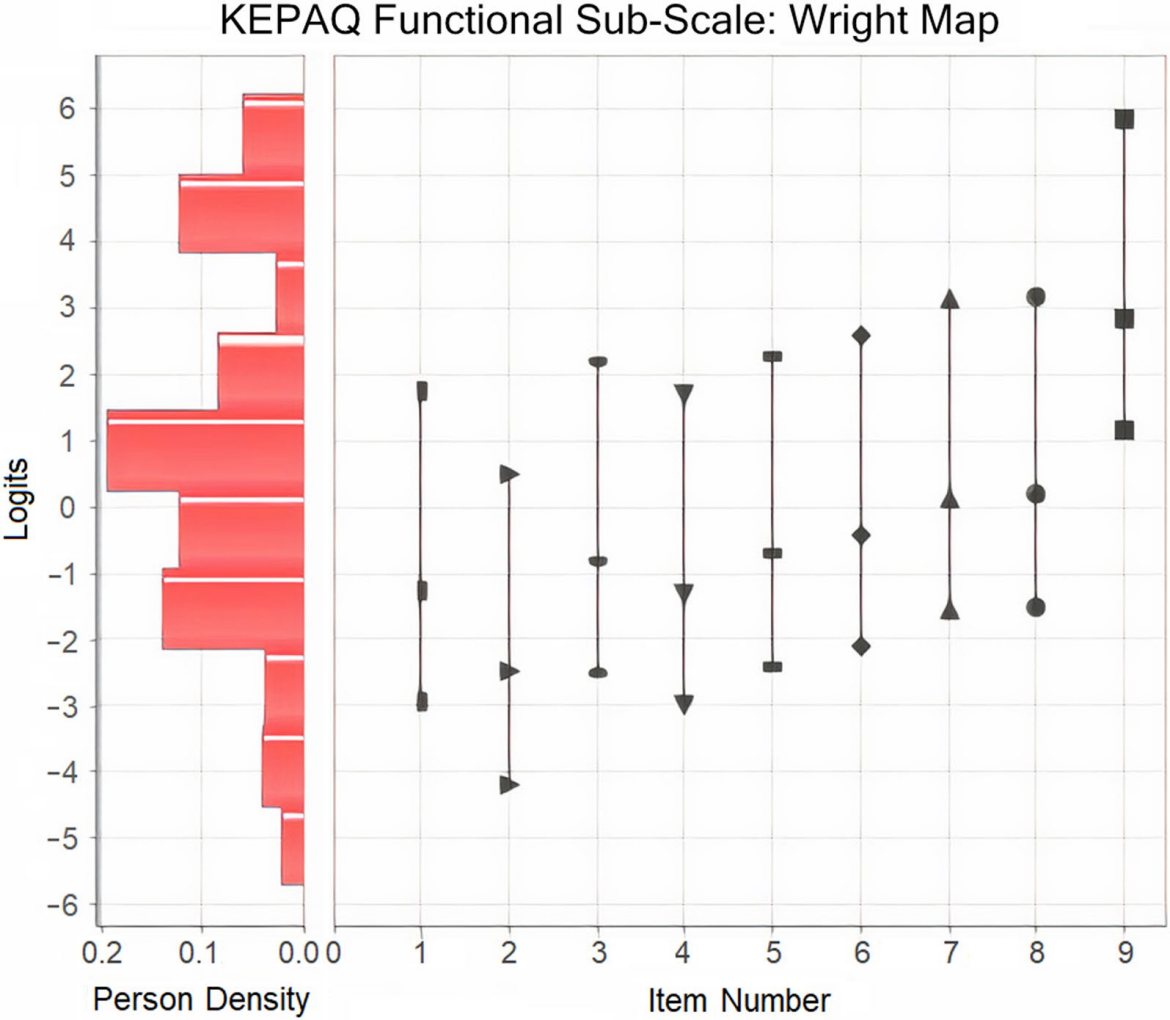
Wright map for the functional compromise sub-scale of the Keratoconus End-Points Assessment Questionnaire (KEPAQ-F)

A Wright map was constructed for the functional sub-scale (Fig. 4).

### KEPAQ scoring

Rasch analysis converts raw scores into a Rasch-derived score ranging from minus infinite to infinite logit, although most measurements fall between − 7.0 and 7.0 logit. A score of 0.0 logits corresponds to a score that is equal to the mean difficulty of the items. Nevertheless, the authors are well aware that this kind of scoring may be difficult to comprehend for authors not dedicated to Rasch studies, and some authors [7] have suggested transforming the score into an easier-to-handle scale. To make results easier to digest for the non-Rasch user, and to standardize the grading of the scale for future international use, the authors have decided to perform a linear transformation ranging from 0 to 100, with a higher score representing a better quality of life.

A table for manually grading the responses of the patient in the KEPAQ-E and KEPAQ-F sub-scales has been developed and are presented in Tables 5 and 6. Using these tables, assign the score for each item corresponding to the response category selected by the patient. Add these scores and divide by the number of questions answered to arrive at the final KEPAQ score in their both sub-scales. To keep standardization, all results from the KEPAQ should be expressed in a 0 to 100 scale. Even when the researchers have obtained a logit value at the first instance, it should be linearly transformed for easy interpretation.

**Table 5:** Table for manually calculating the final score for the emotional compromise sub-scale of the Keratoconus End-Points Assessment Questionnaire (KEPAQ-E)

Question	Not at all	A little	Quite a bit	A lot
Q_E01	76.79	57.98	40.79	25.36
Q_E02	73.27	51.15	36.50	19.68
Q_E03	74.23	57.34	39.67	21.78
Q_E04	73.64	54.94	38.97	19.80
Q_E05	72.92	57.19	37.96	20.08
Q_E06	83.84	70.20	53.20	28.70
Q_E07	82.75	70.50	53.69	31.41

**Table 6:** Table for manually calculating the final score for the functional compromise sub-scale of the Keratoconus End-Points Assessment Questionnaire (KEPAQ-F)

Question	Not at all	A little	Quite a bit	A lot
Q_F01	77.01	52.29	38.14	31.75
Q_F02	76.44	49.98	38.21	15.83
Q_F03	82.18	54.65	40.16	19.35
Q_F04	79.82	52.65	39.45	18.68
Q_F05	83.17	54.83	39.24	20.02
Q_F06	84.76	55.14	40.45	19.79
Q_F07	86.95	57.69	41.15	22.87
Q_F08	86.68	55.85	41.53	26.23
Q_F09	86.16	70.39	48.10	32.42

### KEPAQ classification

So far, no studies have determined the correlation of different scores of the KEPAQ to actual levels of ableness or dis-ableness due to vision. Therefore, it is too early to assign a clear functional classification to different scores of the KEPAQ and this sort of determination is not possible for now.

Currently, the best way of classifying the results of the KEPAQ resides on determining Tukey’s Hinges of the score distribution, and assigning it a denominative ordinal number from 1 to 4 as used in the ABCD classification proposed by Belin for use in keratoconus [5]. For the emotional sub-scale, Tukey’s Hinges are 74.27, 59.15 and 43.90, while for the functional sub-scale they are 69.14, 54.71 and 36.63. Based on these values, a simple table was created for classification (Table 7). Emotional classification should be anteceded by an “E”, so a person within the first and the second Tukey’s Hinge (grade 2) in the emotional sub-scale should be expressed as E2. The same applies with the functional sub-scale but preceded by the letter “F”. We have not included a “0” grading as this denotes an absence of disease [5] and the KEPAQ should only be used in patients with a confirmed diagnosis of keratoconus.

**Table 7:** Table for calculating the E&F classification based on the results of both scales of the Keratoconus End-Points Assessment Questionnaire (KEPAQ)

Grade	Emotional sub-scale (E)	Functional sub-scale (F)
1	≥ 74.27	≥ 69.14
2	59.15–74.26	54.71–69.13
3	43.91–59.14	36.64–54.70
4	≤ 43.90	≤ 36.63

To facilitate computing and classification of KEPAQ measures, we have developed an Excel file that allows the user to easily input the patient’s responses for the scale, obtaining an immediate real-time measure with the corresponding E&F Classification. It allows for a quick and streamlined implementation into everyday practice for researchers and clinicians alike. The Excel file can be freely downloaded from Harvard Dataverse at https://dataverse.harvard.edu/api/access/datafile/4289090.

Please note that we have used the very same system as Belin’s ABCD classification [5], as our ultimate objective was to provide a more complete classification of keratoconus, with categories starting from A to F (ABCDEF). This way, the first three categories (ABC) include anatomical aspects, the fourth category (D) includes functional vision, while the last two letters (EF) comprise emotional and functional quality of life. The first four letters of the classification come from Belin’s paper [5] while the last two letters (EF) come from the present paper. We hope this will provide for a much richer classification of keratoconus to include anatomical, funcional, and subjective aspects of disease.

Our group is currently performing a study using Rasch modeling and machine learning algorithms for better determining the best cut-off values for both sub-scales of the KEPAQ.

## Discussion

Quality of life is an inherently laborious measure for latent trait*.* Part of this difficulty comes from the fact that ‘quality of life’ is a rather abstract concept that encompasses a significant number of characteristics of everyday life, and the correlation of disease to daily functioning.

For decades, it has been highlighted that it is clearly tough to “develop measures of the various dimensions of quality of life while meeting rigorous standards of validity and reliability” [13]. Nevertheless, the last couple of years have seen an increase in the researchers’ perception on the importance of patient-reported outcome measures (PROMs) as a means of better capturing the effect of the health-disease continuum in patients’ day to day activities [14]. Clinicians and researchers have come to notice that the definition of success of management in ophthalmological conditions extends well beyond classical (and easier to measure) variables such as intraocular pressure and visual acuity [15].

Measuring quality of life “is particularly important in keratoconus because it has an early onset, is progressive and chronic in nature and can cause serious vision impairment” [16]. Although some relationship has been demonstrated between corneal distortion and emotional distress [3], it is clear that “the impact of keratoconus on quality of life may be disproportionate to the clinical measures such as best-corrected visual acuity” [16]. Therefore, specifically measuring and classifying quality of life in keratoconus is of top priority if the researchers or clinicians want to have a more complete and holistic picture of the effect the disease has on the patient. This will allow them to have more information than what can be obtained just by measuring simpler characteristics such as corneal distortion or pachymetry.

An accurate measurement of quality of life demands for the use of high-quality and well validated instruments. Kandel et al. very recently reviewed instruments for evaluating quality of life in keratoconus patients, and concluded that there is a need for a comprehensive and psychometrically robust patient-reported outcome measure in keratoconus [16]. Although a number of different scales have been used in corneal ectasia patients, most of them have not being built or validated specifically in keratoconus patients. The problem of using such non-specific scales in different populations from what they were initially designed for, is that results are not necessarily reliable or trustable. So far only two instruments have been specifically created and validated in keratoconus subjects: the KORQ [2, 17–19] and the KEPAQ [1, 3, 6]. Although both scales measure subjective functional compromise by the disease, the main difference between them is the fact that the KEPAQ also has a sub-scale for measuring emotional distress secondary to keratoconus [1]. Measuring emotional well-being has been catalogued as particularly important in keratoconus because the progressive nature of the disease and the possible need of keratoplasty may cause anxiety [16]. Besides, it has been hypothesized that the stage of life at which keratoconus commonly presents plays a crucial role in personality and coping mechanism development that significantly affects behavioral patterns and the relationship with caregivers [20].

This information strongly suggests that measuring both emotional distress and subjective visual functioning may give researchers and clinicians a more complete picture on the burden of disease on keratoconus patients, which is only attainable with the use of the KEPAQ.

Another important aspect to consider is the methods used for scale development and validation. For many decades, previous methods (now collectively known as CTT) were the preponderant means of evaluating the behavior of scales in different populations. Nevertheless, current data has pointed out some very important flaws in the assumptions underlying CTT methods. One of the main drawbacks of CTT is that results created from the mere sum of scores (something now known as “raw scoring”) is erroneously assumed to give an interval-level result, when it is merely ordinal-level in nature. This distinction is important when using parametric statistical analyses for ordinal-level data, which is statistically incorrect and will produce ill results at best. Therefore, conclusions drawn from parametric statistical analyses of ordinal-level data should be taken with a pinch of salt. Moreover, this has been highlighted more than 30 years ago by Merbitz et al. [21] and heartfeltly emphasized in an Editorial by Grimby et al. [22] who mentioned that “perhaps it is time for the academic community to unite in stating enough is enough; that it is time to end the ordinal misrule”, something they have even termed as “malpractice” [22]. The second issue with CTT methods is that they (once again, erroneously) assume that all items should have the same weight towards a final quality of life score. However, some activities will be more limited than others due to disease, so their weights should not be the same while computing a unifying score for measuring quality of life.

To solve both these problems as well as to provide a better way of constructing and evaluating measurement scales, many researchers have increasingly embraced Rasch modeling for their data, bringing about a “revolution that is well underway” [23].

Rasch alleviates many of the ill aspects left about by CTT. It helps transform ordinal-level data into true interval-level data amenable to parametric statistical analyses and conclusions [4]. Besides, it takes into account the non-equal “difficulties” or “calibrations” of the different items that conform to a measuring scale, so the “weight” of every question towards a final score will take into account these differences. “Thus, the Rasch model properties of invariance of comparisons (a given difference meaning the same interval at whatever level of the variable) and sufficiency (the total score implies a predictable score on each of the constituent items, and is all that is required) comply with the axioms of measurement laid down by Luce and Tukey and the latent estimate so derived is an interval scale” [22]. All these reasons provide evidence for the superiority of Rasch modeling when compared to CTT [7].

Nevertheless, some resistance has been felt in the scientific community, partly because non-Rasch researchers may consider Rasch analysis to be non-user-friendly enough, especially for reporting and interpretation. For example, non-scaled results of the KEPAQ have been reported to range between − 5.47 and 6.97 logit [1]. Although this is a totally valid scale for reporting results, numerous authors have indicated that this scale may not be easily digested by non-Rasch practitioners. Therefore, it has been suggested that a linear transformation is performed on the final Rasch person measure to convert it into a positive integer number between 0 and 100 [7]. Our group has deemed appropriate to standardize this linear transformation for reporting the results of the KEPAQ; when providing results in a 0 to 100 scale, a higher number translates to a better quality of life.

To further facilitate computation of the KEPAQ, in the present article we have provided two different means of calculating the final score in an interval-level means. First, a “manual” way in which a table is used to compute every response and then the relative eight of every question is averaged to arrive at the final KEPAQ score. This method has been proven to be better than what our group had already published (raw score to final score) [1] as this allows for computation in the event of missing responses and better respects the different relative weights of every question towards the scale score. Second, a very user-friendly Excel file has been constructed, which allows the tester to input responses and obtain real-time calculation of the final score and classification of the KEPAQ in the E&F classification. The file can downloaded from Harvard Dataverse from the given link.

An element we feel interesting on our paper is that we suggest a way in which quality of life can be easily adapted to current ABCD Belin classification [5] for constructing a classification system that better represents the whole experience of having keratoconus (corneal distortion, visual acuity, and quality of life). The authors have intended for the E&F part of the classification to be easily understandable and implemented, following the very same denomination and expression used for the ABCD classification. Besides, the KEPAQ has been demonstrated to correlate well with ABCD classification in the worse eye [3], so there is potential for both classifications to work well together. Through the implementation of simple scale measurement (which can be easily filled up by the patient in the waiting room) and the Excel file attached, the authors feel that implementation of this classification should be very straightforward for the clinician, without increasing time spent with the patient, and providing a better picture of how disease and treatment impacts the patient. As time passes and more patients are evaluated using the KEPAQ, distribution of Tukey’s Hinges will be improved, allowing for a more accurate determination of the E&F classification in different populations.

## Conclusions

Measurement of the quality of life in keratoconus patients is of paramount importance for both the ophthalmology community. So far, the only scale for measuring both functional and emotional compromise specifically designed for keratoconus patients is the KEPAQ. This scale has been demonstrated to comply with the “specially demanding” [22] Rasch model expectations, so the authors can be confident that KEPAQ is able to adequately measure quality of life in these patients. In this paper, we proposed a means for standardizing the calculation and reporting KEPAQ results, as well as a classification method that could be implemented along with the Belin ABCD Classification [5], and thus provide a method to better capture the current state of patients with keratoconus.

## Data Availability

The authors of this study believe in public availability of research data for re-analysis, data mining and for securing the transparency of research. The database used for the analysis of this paper is freely and permanently available from Harvard Dataverse at https://doi.org/10.7910/DVN/APBOX9.
